# Malnutrition and sarcopenia: a combined risk factor for vascular calcification and cardiovascular events in hemodialysis patients

**DOI:** 10.3389/fnut.2025.1625935

**Published:** 2025-11-14

**Authors:** Zitao Wang, Lu Jiang, Qing Li, Rui Xu, Yanggang Yuan, Huijuan Mao, Changying Xing

**Affiliations:** 1 Department of Nephrology, The First Affiliated Hospital with Nanjing Medical University, Nanjing Medical University, Nanjing, China; 2 Department of Nephrology, Jiangdu People’s Hospital Affiliated to Yangzhou University, Yangzhou, China

**Keywords:** malnutrition, sarcopenia, hemodialysis, vascular calcification, major adverse cardiovascular events, chronic kidney disease

## Abstract

**Background:**

Hemodialysis (HD) patients are at high risk of vascular calcification (VC) and cardiovascular disease (CVD). The coexistence of sarcopenia and malnutrition, known as malnutrition-sarcopenia syndrome (MSS), may further exacerbate these risks. This study aimed to investigate the prevalence of MSS in HD patients and its association with abdominal aortic calcification (AAC) and major adverse cardiovascular events (MACE).

**Methods:**

This prospective cohort study enrolled 462 maintenance HD patients, who were subsequently stratified into four groups: no sarcopenia-no malnutrition, sarcopenia alone, malnutrition alone, and MSS. Sarcopenia was diagnosed based on the 2019 Asian Working Group for Sarcopenia (AWGS) criteria, and malnutrition was assessed using the Geriatric Nutritional Risk Index (GNRI). AAC was evaluated using lateral abdominal radiographs and scored according to Kauppila’s semiquantitative scoring system. The primary outcome was MACE during a 3-year follow-up period. Multivariable logistic regression and mediation analyses were employed to determine associations and potential mechanisms.

**Results:**

The prevalence of sarcopenia and malnutrition was 46.3 and 32.0%, respectively, with 18.2% of patients having MSS. MSS was independently associated with increased AAC (OR: 2.157, 95% CI: 1.064–4.373, *p* = 0.033) and MACE risk (OR: 2.235, 95% CI: 1.192–4.194, *p* = 0.012). Mediation analysis revealed that AAC severity partially mediated the relationship between MSS and MACE, accounting for 26.7% of the total effect.

**Conclusion:**

MSS is prevalent in HD patients and is associated with more severe VC and higher cardiovascular risk. Comprehensive nutritional assessment and targeted interventions are needed to address sarcopenia and malnutrition in HD patients to improve their cardiovascular outcomes.

## 1 Introduction

Hemodialysis (HD) is a primary renal replacement therapy for end-stage renal disease (ESRD) caused by chronic kidney disease (CKD). Cardiovascular disease (CVD) is the leading cause of mortality in HD patients. These patients face a 50% higher overall risk of developing CVD compared to the general population (1). Moreover, once CVD is present, their CVD-related mortality rate is 20-fold higher (2). Vascular calcification (VC), a key driver of CVD in ESRD, affects 80–90% of CKD patients (3). Early detection and intervention to mitigate VC-associated cardiovascular risks in this population remain critical research priorities.

Sarcopenia is a progressive and generalized skeletal muscle disorder characterized by reduced muscle mass, strength, and physical performance. HD patients frequently develop sarcopenia due to chronic inflammation, metabolic acidosis, protein-energy wasting (PEW), and physical inactivity (4, 5). The prevalence of sarcopenia in HD patients ranges from 4 to 68%, with an overall prevalence of 28.5% (5). Meta-analyses indicate a strong association between sarcopenia and cardiovascular events in dialysis patients (6). Additionally, malnutrition is a major risk factor in CKD patients, contributing to poor clinical outcomes (7). It is commonly defined as “a state resulting from lack of intake or uptake of nutrition that leads to altered body composition (decreased fat free mass) and body cell mass leading to diminished physical and mental function and impaired clinical outcome from disease” (8). There is substantial overlap between sarcopenia and malnutrition, leading Vandewoude et al. (9) to propose the malnutrition-sarcopenia syndrome (MSS), defined as the co-occurrence of malnutrition and sarcopenia. MSS confers a higher mortality risk than either condition alone (10, 11). Despite its clinical significance, research on MSS in HD populations remains limited. Current studies suggest that MSS increases mortality risk in HD patients (12–14), but the underlying mechanisms remain unclear. Crucially, it is unknown whether MSS further exacerbates CVD and VC risk in this high-risk population.

The abdominal aortic calcification (AAC) score has emerged as a widely used quantitative tool for assessing VC severity in HD patients due to its noninvasive nature, operational simplicity, and reproducibility. Building upon these findings, we conducted this study to investigate whether HD patients with MSS exhibit higher AAC prevalence and face increased risks of major adverse cardiovascular events (MACE). Our findings may enhance clinicians’ awareness of these critical associations and underscore the importance of addressing both sarcopenia and malnutrition in routine HD care.

## 2 Materials and methods

### 2.1 Study design and participants

This prospective cohort study enrolled patients receiving HD between January 2021 and February 2022. Inclusion criteria were: (1) stable HD treatment for >6 months (3 sessions/week; 4 h/session) and (2) age ≥18 years. Exclusion criteria were: (1) Active comorbidities: malignancy, severe infection within the past 3 months, or heart failure (NYHA Class II or higher); (2) Limb mobility impairment preventing sarcopenia assessment (e.g., inability to perform grip strength or gait speed tests); (3) History of orthopedic surgery involving the lower limbs within the past 3 months. The study protocol was approved by the Institutional Review Board of our hospital (YJRY-2020-K-024, 2019-SR-368). All patients provided written informed consent and completed the study with no loss to follow-up. Follow-up was conducted through regular outpatient visits during hemodialysis sessions and telephone interviews during interdialytic periods. Clinical events occurring outside the study center were captured via patient/family reporting and verification through external medical records.

### 2.2 Treatment protocol

All patients received standard care comprising maintenance HD, pharmacological therapy, nutritional support, and psychological counseling. Comorbid conditions including anemia, electrolyte imbalances, chronic kidney disease and mineral bone disorders (CKD-MBD) were managed according to established clinical guidelines (15, 16). Hypertension, diabetes, and dyslipidemia were managed through appropriate medical interventions. Malnourished patients received individualized nutritional support according to a standardized institutional protocol, detailed in Supplementary Table 1. This protocol was developed in alignment with contemporary international guidelines (17). In brief, the protocol included dietary counseling, oral nutritional supplements (ONS), and/or intradialytic parenteral nutrition (IDPN) based on the severity of malnutrition and the patient’s ability to meet nutritional requirements orally. Adherence to ONS was monitored weekly through patient interviews and pill counts of provided supplements. Adherence to IDPN was 100% as it was administered during dialysis sessions. Psychological counseling was provided to address mental health issues and improve overall well-being, while patients were encouraged to engage in physical exercise appropriate to their functional capacity and health status.

### 2.3 Clinical parameters

We prospectively collected comprehensive baseline characteristics including demographic data (gender, age), dialysis duration, urea clearance index (Kt/V), blood pressure, body mass index (BMI), and comorbidities, along with laboratory parameters: complete blood count, liver/renal function tests, C-reactive protein (CRP), neutrophil-to-lymphocyte ratio (NLR), platelet-to-lymphocyte ratio (PLR), lipid profile, parathyroid hormone (PTH) levels, iron indices, electrolyte panel, and β2-microglobulin measurements. Physical activity level was assessed using the short form of the International Physical Activity Questionnaire (IPAQ) and categorized into low, moderate, and high levels according to standard scoring guidelines (18, 19). Based on the presence of sarcopenia and malnutrition, participants were stratified into four groups: (1) no sarcopenia-no malnutrition; (2) sarcopenia alone; (3) malnutrition alone; and (4) MSS group.

### 2.4 AAC assessment

AAC scores were determined using lateral abdominal radiographs evaluated by two blinded radiologists employing Kauppila’s semiquantitative scoring system (20). Inter- and intra-observer agreement for AAC scoring was assessed using intraclass correlation coefficient (ICC >0.89) and Cohen’s kappa (*κ* > 0.85). Discrepancies (<11% of cases) were resolved by a third independent radiologist. The anterior and posterior aortic walls adjacent to each lumbar vertebra (L1–L4) were graded: 0 (no calcification), 1 (small scattered deposits covering <1/3 vertebral length), 2 (deposits covering 1/3–2/3 length), or 3 (deposits >2/3 length). Total scores (range 0–24) were calculated by summing all segmental scores, with final scores representing the average of both readers’ assessments. Participants were stratified by AAC severity: ≤5 (non-calcified group) versus >5 (calcified group).

### 2.5 Sarcopenia diagnosis

Sarcopenia was diagnosed according to the 2019 Asian Working Group for Sarcopenia (AWGS) criteria (21), requiring: (1) low muscle strength (handgrip strength <28 kg for men or <18 kg for women); (2) poor physical performance (6-m gait speed <1.0 m/s); and (3) low muscle mass [(appendicular skeletal muscle mass index, ASMI) <7.0 kg/m^2^ for men or <5.7 kg/m^2^ for women]. Diagnosis required confirmation of low muscle mass (criterion 3) plus either criterion 1 or 2.

### 2.6 Muscle strength and physical performance assessment

Following AWGS 2019 protocols (21), muscle strength was assessed using a calibrated digital handgrip dynamometer (EH101 model, Xiangshan Instruments, Guangdong, China) in accordance with AWGS protocols. Physical performance was assessed via 6-m gait speed test performed twice with averaged results. Muscle mass was measured using a bioelectrical impedance analysis (BIA) (InbodyS10, InBody Co., Ltd., Seoul, Korea). Standardized patient preparation and positioning protocols were employed to ensure measurement reliability.

### 2.7 Nutritional assessment

The Geriatric Nutritional Risk Index (GNRI), a well-validated nutritional screening tool with demonstrated prognostic value in hemodialysis populations (22), was employed as a comprehensive nutritional indicator. Although GNRI is a risk screening tool, a score <92 is widely accepted in the literature to indicate malnutrition in hemodialysis patients. GNRI was calculated using the following formula (23): GNRI = [1.489 × serum albumin (g/L)] + [41.7 × (current body weight/ideal body weight)]. Individuals with a GNRI score <92 were categorized as having malnutrition (24).

### 2.8 Study outcomes

During the 3-year follow-up period, the primary endpoint was MACE, defined as a composite of cardiovascular mortality or hospitalization due to acute myocardial infarction, acute heart failure, or stroke.

### 2.9 Statistical analysis

*A priori* sample size calculation was performed based on expected survival rates from prior literature (12): 86.7% (no sarcopenia-no malnutrition), 74.2% (sarcopenia alone), 84.5% (malnutrition alone), and 68.9% (MSS), with a dropout rate of 5%, *α* = 0.05, and power = 80%. The minimum required sample size was 431. Our final cohort of 462 patients exceeded this requirement. All analyses were performed using SPSS 27.0 (IBM Corp.). Categorical variables were expressed as frequencies and percentages. Normally distributed continuous variables were presented as mean ± SD and compared using Student’s *t*-test, while non-normally distributed data were expressed as median (Q1, Q3) and analyzed with the Mann–Whitney *U* test. Multivariable logistic regression identified independent risk factors for vascular calcification. Kaplan–Meier analysis with the log-rank test assessed the probability of survival of MACE and all-cause mortality. A mediation analysis was performed using SPSS PROCESS macro (Model 4). The Bootstrap method was employed to evaluate the significance of the indirect effect statistical significance was set at *p <* 0.05

## 3 Results

### 3.1 Study participants

**Figure 1:**
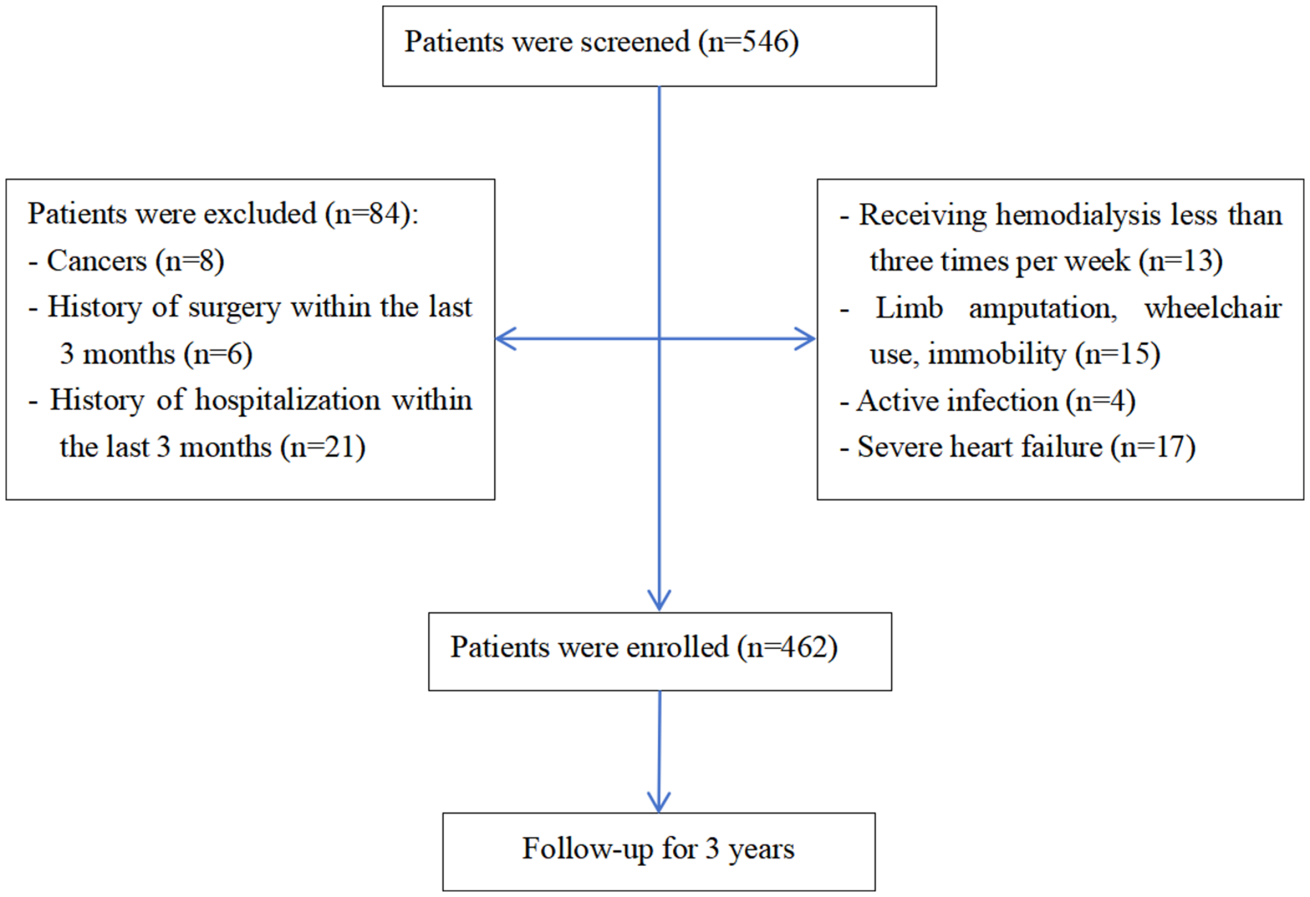
Study flowchart.

**Figure 2:**
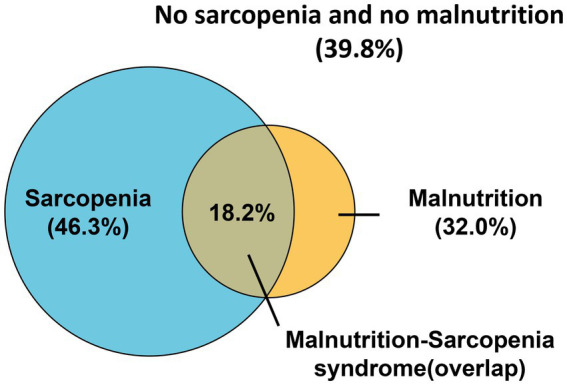
Venn diagram of patients with sarcopenia, malnutrition, and malnutrition-sarcopenia syndrome.

**Figure 3:**
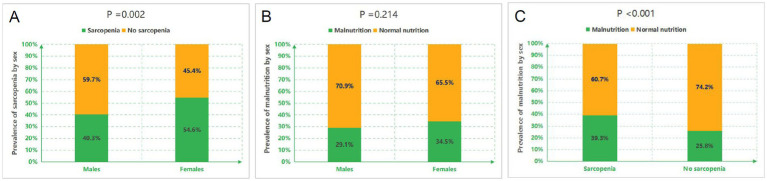
Prevalence of sarcopenia and malnutrition across subgroups. **(A)** Gender-specific prevalence of sarcopenia. **(B)** Gender-specific prevalence of malnutrition. **(C)** Prevalence of malnutrition stratified by sarcopenia status.

Of 546 initially screened HD patients, 84 were excluded per eligibility criteria, yielding 462 enrolled participants (Figure 1). The cohort comprised 268 males (58.0%) and 194 females (42.0%) with a mean age of 57.13 ± 12.06 years. The prevalence of sarcopenia was 46.3% overall (214/462), with significant gender disparity [males: 40.3% (108/268) vs. females: 54.6% (106/194); *χ*^2^ = 9.308, *p* = 0.002] (Figure 2). Malnutrition was identified in 32.0% (148/462), with no significant gender difference [males: 29.1% (78/268) vs. females: 34.5% (67/194); *χ*^2^ = 1.542, *p* = 0.214]. Sarcopenic patients had a higher prevalence of malnutrition than non-sarcopenic counterparts [39.3% (84/214) vs. 25.8% (64/248); *p <* 0.001, Figure 3]. MSS was present in 18.2% (84/462, Figure 2).

### 3.2 Baseline characteristics

HD patients were stratified into four groups: (1) no sarcopenia-no malnutrition (*n* = 184); (2) Sarcopenia alone (*n* = 130); (3) Malnutrition alone (*n* = 64); and (4) MSS (*n* = 84). Table 1 presents detailed comparisons of baseline characteristics, muscle parameters, and AAC status. Chronic glomerulonephritis (58.4%) and diabetic nephropathy (22.5%) were the predominant primary renal diseases.

**Table 1:** Characteristics from HD patients with and without sarcopenia or malnutrition.

Characteristics	All	No sarcopenia-no malnutrition	Sarcopenia alone	Malnutrition alone	MSS	*p-*value
No. of participants (*n*)	462	184	130	64	84	
Clinical data						
Gender, male (*n*)	268 (58.0)	130 (70.7)	60 (46.2)	30 (46.9)	48 (57.1)	0.002
Age (years)	57.13 ± 12.06	51.61 ± 9.34	61.54 ± 12.31	53.81 ± 11.81	64.93 ± 10.32	<0.001
Diabetes [*n* (%)]	114 (24.7)	34 (18.5)	44 (33.8)	12 (18.8)	24 (28.6)	0.009
Hypertension [*n* (%)]	332 (71.9)	36 (73.9)	94 (72.3)	42 (65.6)	60 (71.4)	0.652
ESRD primary cause [*n* (%)]						0.778
Glomerulonephritis	270 (58.4)	118 (64.1)	62 (47.7)	40 (62.5)	50 (59.5)	
Diabetes mellitus	104 (22.5)	34 (18.5)	38 (29.2)	12 (8.8)	20 (23.8)	
Polycystic nephropathy	26 (5.6)	8 (4.3)	12 (9.2)	4 (6.3)	2 (2.4)	
Hypertensive nephropathy	36 (7.8)	14 (7.6)	12 (9.2)	6 (9.4)	4 (4.8)	
Other	26 (5.6)	10 (5.4)	6 (4.6)	2 (3.1)	8 (9.5)	
Vascular access [*n* (%)]						<0.001
Catheter	66 (14.3)	14 (7.6)	30 (23.1)	6 (9.4)	16 (19.0)	
Arteriovenous fistula	396 (85.7)	170 (92.4)	100 (76.9.)	58 (90.6)	68 (81.0)	
Physical activity leve l (IPAQ) [*n* (%)]						0.166
High level	234 (50.7)	101 (54.9)	62 (47.7)	32 (50.0)	39 (46.4)	
Moderate level	135 (29.2)	57 (31.0)	35 (26.9)	19 (29.7)	24 (28.6)	
Low level	93 (20.1)	26 (14.1)	33 (25.4)	13 (20.3)	21 (25.0)	
Medications [*n* (%)]						
Calcium carbonate	146 (31.8)	54 (29.3)	44 (33.8)	22 (19.4)	26 (31.0)	0.729
Erythropoietin	386 (84.1)	158 (85.9)	106 (81.5)	54 (88.5)	68 (81.0)	0.460
Iron supplements	64 (13.9)	27 (25.7)	19 (14.6)	8 (13.1)	10 (11.9)	0.928
Vitamin D	170 (37.0)	72 (39.1)	50 (38.5)	22 (36.1)	26 (31.0)	0.611
Statins	69 (15.0)	31 (16.8)	20 (15.4)	8 (13.1)	10 (11.9)	0.728
Non-calcium phosphate binders	144 (31.4)	52 (28.3)	48 (36.9)	20 (32.8)	24 (28.6)	0.384
Laboratory data						
Hemoglobin (g/L) (120–160)	110.03 ± 22.11	110.7 ± 21.88	110.25 ± 20.96	109.47 ± 25.72	108.67 ± 21.75	0.911
Albumin (g/L) (35–52)	40.3 ± 3.57	42.66 ± 2.42	41.99 ± 2.88	36.48 ± 3.51	35.42 ± 3.66	<0.001
Blood urea nitrogen (mmol/L) (2.76–8.07)	25.33 ± 5.78	26.26 ± 6.19	24.96 ± 5.24	24.62 ± 6.17	24.41 ± 5.08	0.038
Serum creatinine (μmol/L) (45–104)	899.85 ± 218.58	991.27 ± 219.76	865.73 ± 185.52	868.84 ± 190.61	776.04 ± 202.97	<0.001
Serum urea acid (μmol/L) (202.3–416.5)	434.63 ± 116.18	431.09 ± 103.6	439.3 ± 148.8	434.03 ± 102.86	435.64 ± 93.84	0.943
Serum magnesium (mmol/L) (0.65–1.05)	1.19 ± 0.17	1.2 ± 0.18	1.19 ± 0.16	1.21 ± 0.18	1.15 ± 0.13	0.071
Serum phosphorus (mmol/L) (0.87–1.45)	2.17 ± 0.64	2.29 ± 0.66	2.21 ± 0.61	2.07 ± 0.54	1.92 ± 0.64	0.004
Serum calcium (mmol/L) (2.15–2.55)	2.3 ± 0.22	2.3 ± 0.21	2.35 ± 0.26	2.3 ± 0.18	2.24 ± 0.21	<0.001
Calcium-phosphorus product (mmol^2^/L^2^) (<4.4)	5.01 ± 1.63	5.28 ± 1.67	5.2 ± 1.59	4.78 ± 1.34	4.3 ± 1.58	<0.001
TG (mmol/L) (0–1.7)	1.56 (1.04, 2.40)	1.72 (1.18, 2.82)	1.71 (1.22, 2.44)	1.46 (0.93, 2.06)	1.15 (0.87, 1.58)	<0.001
TC (mmol/L) (2.0–5.7)	3.53 ± 0.85	3.56 ± 0.84	3.56 ± 0.86	3.82 ± 0.94	3.18 ± 0.72	<0.001
BMI (kg/m^2^)	22.89 ± 3.76	24.34 ± 3.54	23.9 ± 3.07	19.81 ± 3.93	20.52 ± 2.29	<0.001
Kt/V	1.42 ± 0.22	1.41 ± 0.25	1.41 ± 0.18	1.43 ± 0.24	1.45 ± 0.22	0.649
Dialysis vintage (months)	69.00 (34.00, 123.00)	66.50 (32.00, 113.75)	68.00 (39.00, 112.25)	89.00 (40.25, 144.50)	70.50 (30.00, 126.00)	0.134
PLR	136.34 (103.76, 180.16)	133.05 (106.63, 166.88)	130.77 (102.33, 196.40)	153.93 (118.48, 223.01)	140.83 (100.41, 186.48)	0.087
NLR	3.59 (2.51, 5.02)	3.64 (2.54, 4.54)	3.74 (2.76, 5.59)	3.50 (2.32, 5.18)	3.42 (2.38, 5.78)	0.308
CRP (mg/L) (0–10)	1.94 (0.50, 5.12)	1.47 (0.50, 4.37)	2.62 (0.50, 6.00)	1.46 (0.50, 3.90)	2.99 (15.10, 7.45)	0.002
PTH (pg/mL) (15–65)	185.00 (79.80, 289.00)	184.50 (75.13, 283.00)	201.00 (84.65, 309.50)	213.50 (84.85, 416.25)	130.00 (54.98, 242.50)	0.046
Transferrin saturation (%) (20–55)	30.00 (21.00, 39.00)	30.50 (22.00, 40.75)	31.00 (25.00, 36.00)	27.00 (20.00, 46.00)	28.50 (16.75, 38.25)	0.224
Serum ferritin (ng/mL) (30–410)	127.50 (43.00, 368.00)	110.50 (49.30, 286.73)	168.80 (45.50, 462.50)	72.70 (26.64, 359.50)	143.10 (41.69, 600.00)	0.038
Total iron-binding capacity (μmol/L) (50–77)	49.73 ± 11.11	52.1 ± 10.68	48.1 ± 11.09	50.55 ± 12.37	46.42 ± 9.92	<0.001
Serum iron (μmol/L) (10.6–36.7)	13.70 (11.00, 17.80)	15.25 (12.00, 19.53)	13.50 (11.25, 17.00)	13.65 (10.33, 18.98)	11.75 (8.50, 14.73)	<0.001
25-(OH) vitamin D (ng/mL) (>20)	19.83 (15.25, 26.61)	24.14 (17.28, 30.64)	18.60 (13.25, 23.79)	22.86 (15.78, 27.81)	17.24 (12.49, 20.41)	<0.001
β2-microglobulin (mg/L) (1–3)	36.97 ± 10.52	36.47 ± 10.76	37.51 ± 9.37	33.6 ± 10.78	39.77 ± 10.83	0.004
Handgrip strength (kg)	24.20 (18.60, 31.08)	14.00 (9.58, 18.05)	19.05 (11.03, 26.60)	12.65 (6.80, 17.80)	17.90 (12.00, 25.90)	<0.001
Gait speed (m/s)	0.94 ± 0.23	1.04 ± 0.2	0.85 ± 0.23	1.03 ± 0.17	0.8 ± 0.19	<0.001
ASMI (kg/m^2^)	7.18 ± 2.41	8.95 ± 2.01	5.36 ± 0.79	8.59 ± 2.01	5.07 ± 1.04	<0.001
GNRI	99.54 ± 8.5	105.91 ± 6.82	104.83 ± 5.52	87.64 ± 4.96	86.48 ± 4.87	<0.001
AAC score	7.00 (1.00, 13.00)	4.00 (0.00, 10.75)	9.00 (2.00, 17.00)	5.00 (0.00, 9.00)	10.00 (4.50, 19.00)	<0.001
AAC [*n* (%)]	254 (55.0)	78 (42.4)	86 (66.2)	28 (43.8)	62 (73.8)	<0.001

MSS, malnutrition-sarcopenia syndrome; ESRD, end-stage renal disease; NLR, neutrophil-to-lymphocyte ratio; PLR, platelet-to-lymphocyte ratio; TG, triglycerides; TC, total cholesterol; BMI, body mass index; Kt/V, urea clearance index; CRP, C-reactive protein; PTH, parathyroid hormone; ASMI, appendicular skeletal muscle mass index; GNRI, Geriatric Nutritional Risk Index; AAC, abdominal aortic calcification. IPAQ, International Physical Activity Questionnaire.

Significant intergroup differences emerged in gender distribution and age: the MSS group was predominantly male (vs. female predominance in the no sarcopenia-no malnutrition group) and had the oldest participants (followed by the sarcopenia-alone group). These two groups also showed higher prevalence of diabetes and catheter use compared to other groups.

### 3.3 Biochemical and functional parameters

Significant intergroup differences were observed in biochemical profiles and functional parameters. The MSS group demonstrated the lowest serum albumin and creatinine levels, along with reduced calcium-phosphate product, PTH, triglycerides (TG), and total cholesterol (TC) concentrations. Additionally, this group exhibited the lowest total iron-binding capacity, serum iron, and vitamin D levels, but the highest β2-microglobulin values. Compared to both the no sarcopenia-no malnutrition and sarcopenia-alone groups, MSS patients had significantly lower BMI (all *p <* 0.05).

Marked variations were noted in muscle function and nutritional indices across groups. Handgrip strength, gait speed, ASMI, and GNRI all showed significant intergroup differences (*p <* 0.01). The MSS group presented with the highest CRP levels, followed by the sarcopenia-alone group. Both MSS and sarcopenia-alone groups had elevated ferritin levels compared to the no sarcopenia-no malnutrition and malnutrition-alone groups (*p <* 0.05).

### 3.4 Vascular calcification patterns

Overall AAC prevalence (score >5) was 55.0% in the total cohort. Group-specific prevalence rates demonstrated significant variation (*p <* 0.001): 42.4% in no sarcopenia-no malnutrition, 66.2% in sarcopenia-alone, 43.8% in malnutrition-alone, and 73.8% in MSS groups, with the highest occurrence observed in MSS patients (Table 1). To further compare AAC prevalence among the four groups, pairwise chi-square tests with Bonferroni correction (significance set at *p* < 0.0083) were performed. The MSS group showed significantly higher AAC prevalence than both the no sarcopenia-no malnutrition and malnutrition alone groups (both *p* < 0.001), but not the sarcopenia alone group. The sarcopenia alone group had higher AAC prevalence than the no sarcopenia-no malnutrition and malnutrition alone groups (*p* < 0.001 and *p* = 0.002, respectively). No significant difference was found between the malnutrition alone and no sarcopenia-no malnutrition groups.

**Figure 4:**
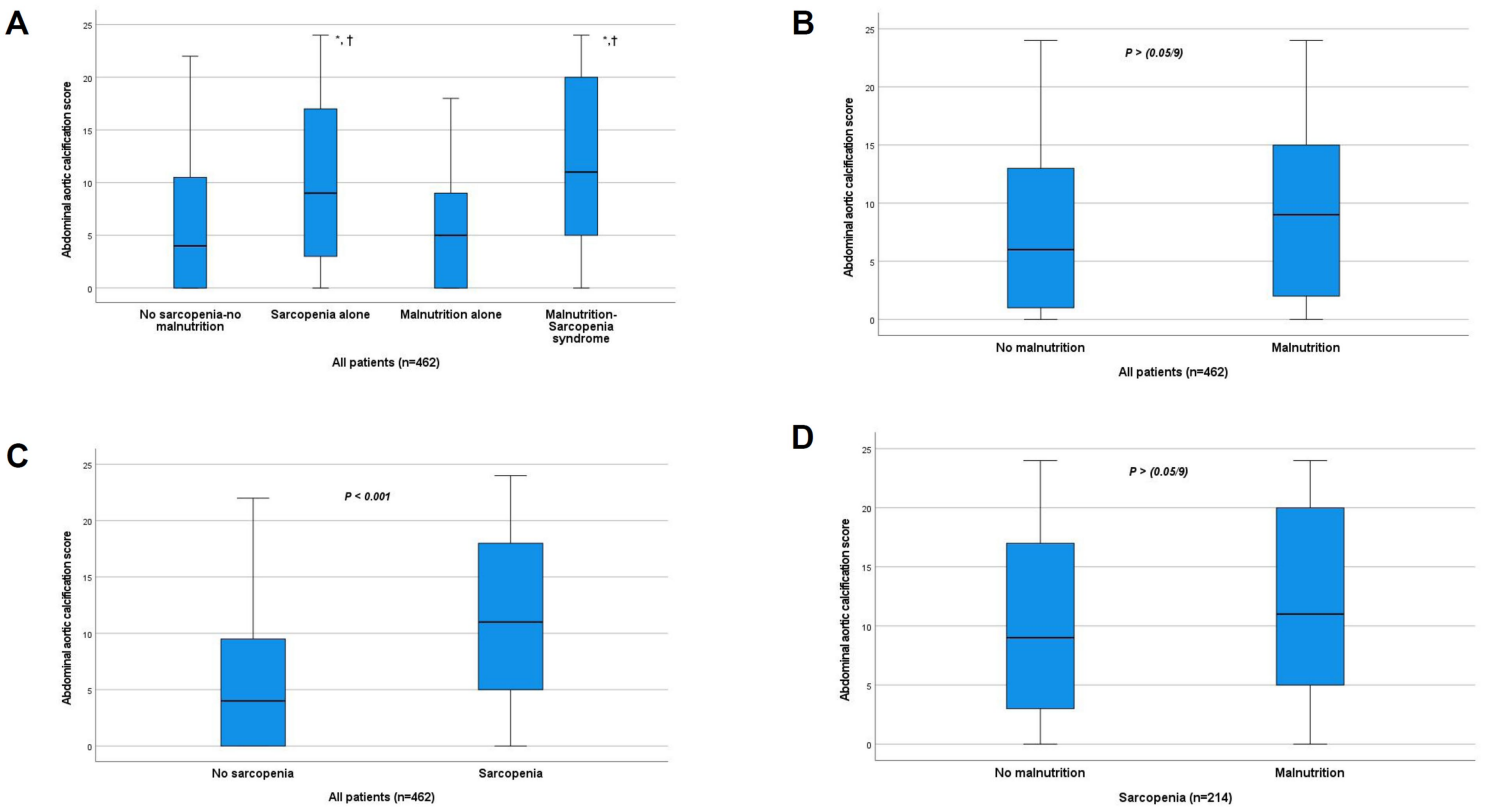
Comparison of abdominal aortic calcification score between groups. **(A)** Comparison between the four study groups. ^*^p < 0.001 vs. no sarcopenia-no malnutrition group. ^†^*p* < 0.001 vs. malnutrition-alone group (all significant after Bonferroni correction, *α* < 0.0056). The difference between MSS and Sarcopenia alone groups was not significant after correction. **(B)** Comparison by nutritional status (*p* > 0.0056, NS after Bonferroni correction). **(C)** Comparison by sarcopenia status (*p* < 0.001). **(D)** Comparison by nutritional status within the sarcopenic subgroup (*p* > 0.0056, NS after Bonferroni correction). NS, not significant.

Quantitative analysis revealed significant differences in AAC scores among groups (*p* < 0.001). After Bonferroni correction (significance level set at *p* < 0.0056), the MSS group had significantly higher AAC scores than the no sarcopenia-no malnutrition group and the malnutrition-alone group (both *p* < 0.001), but not the sarcopenia-alone group. The sarcopenia-alone group had significantly higher scores than the no sarcopenia-no malnutrition group and the malnutrition-alone group (both *p* < 0.001). No significant difference was found between the malnutrition-alone and no sarcopenia-no malnutrition groups (Figure 4A). When stratified by nutritional status, the difference in AAC scores between malnourished [9.00 (2.00, 15.00)] and non-malnourished patients [6.00 (1.00, 13.00)] was not statistically significant after Bonferroni correction (*p* > 0.0056) (Figure 4B). Sarcopenic patients had significantly higher scores [11.00 (4.75–18.00)] than non-sarcopenic individuals [4.00 (0.00, 9.75), p < 0.001] (Figure 4C). Within the sarcopenic subgroup, the difference in AAC scores between those with concomitant malnutrition [11.00 (5.00, 20.00)] and those without [9.00 (2.50, 17.00)] was also not statistically significant after Bonferroni correction (*p* > 0.0056) (Figure 4D).

### 3.5 Correlation analysis

**Figure 5:**
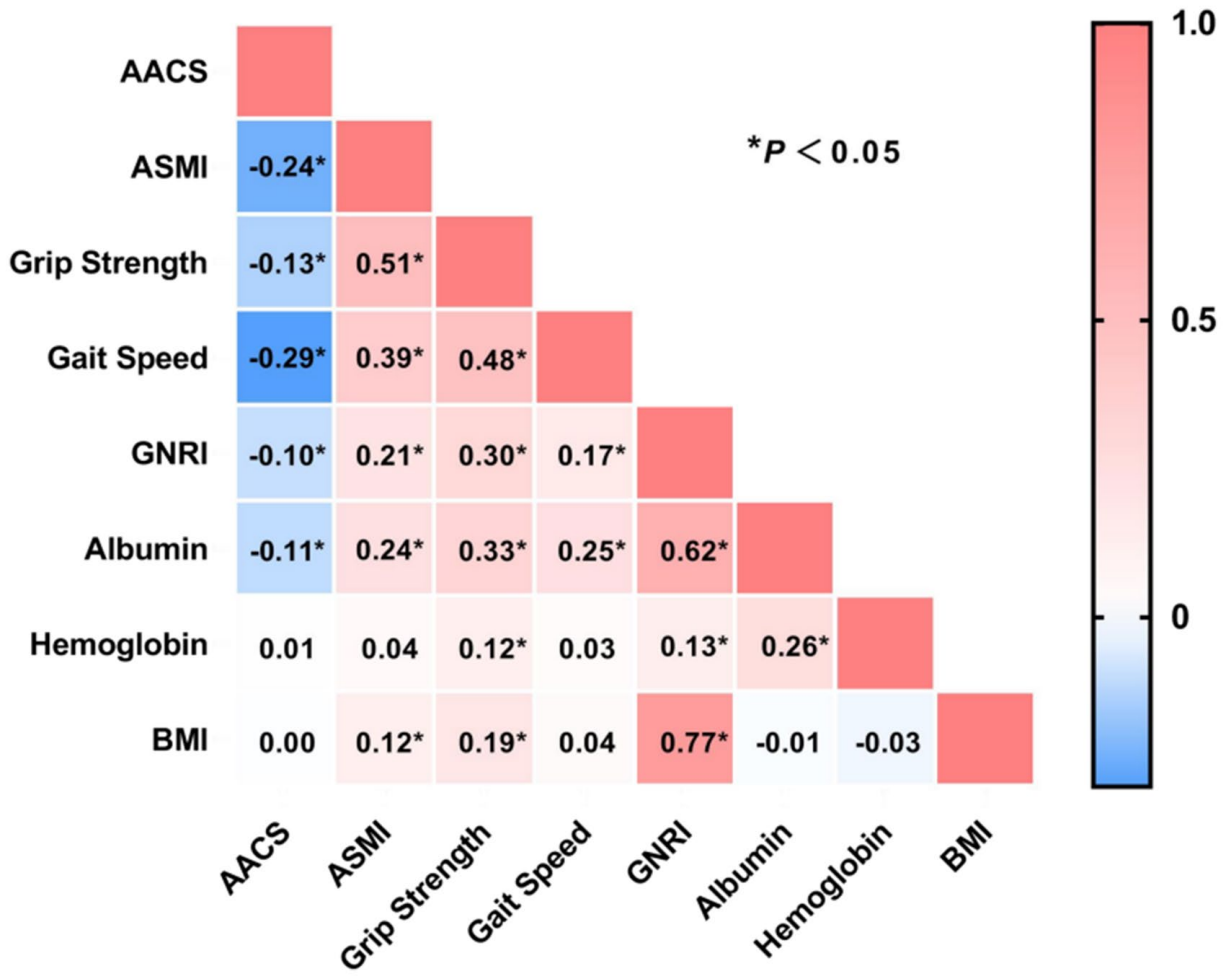
Correlation analysis between abdominal aortic calcification score with sarcopenia and nutritional markers. AACS, abdominal aortic calcification score; ASMI, appendicular skeletal muscle index; GNRI, Geriatric Nutritional Risk Index; BMI, body mass index. ^*^p < 0.05.

To evaluate the clinical relationship between AAC degree and MSS, we performed a correlation analysis between the AAC score and MSS-related clinical indicators. The results indicated that AAC score was negatively correlated with ASMI, grip strength, gait speed, GNRI, and serum albumin levels (all *p*-values <0.05). In contrast, no significant correlations were found between AAC score and hemoglobin levels or BMI (Figure 5).

### 3.6 Kaplan–Meier survival analysis for MACE and all-cause mortality

**Figure 6:**
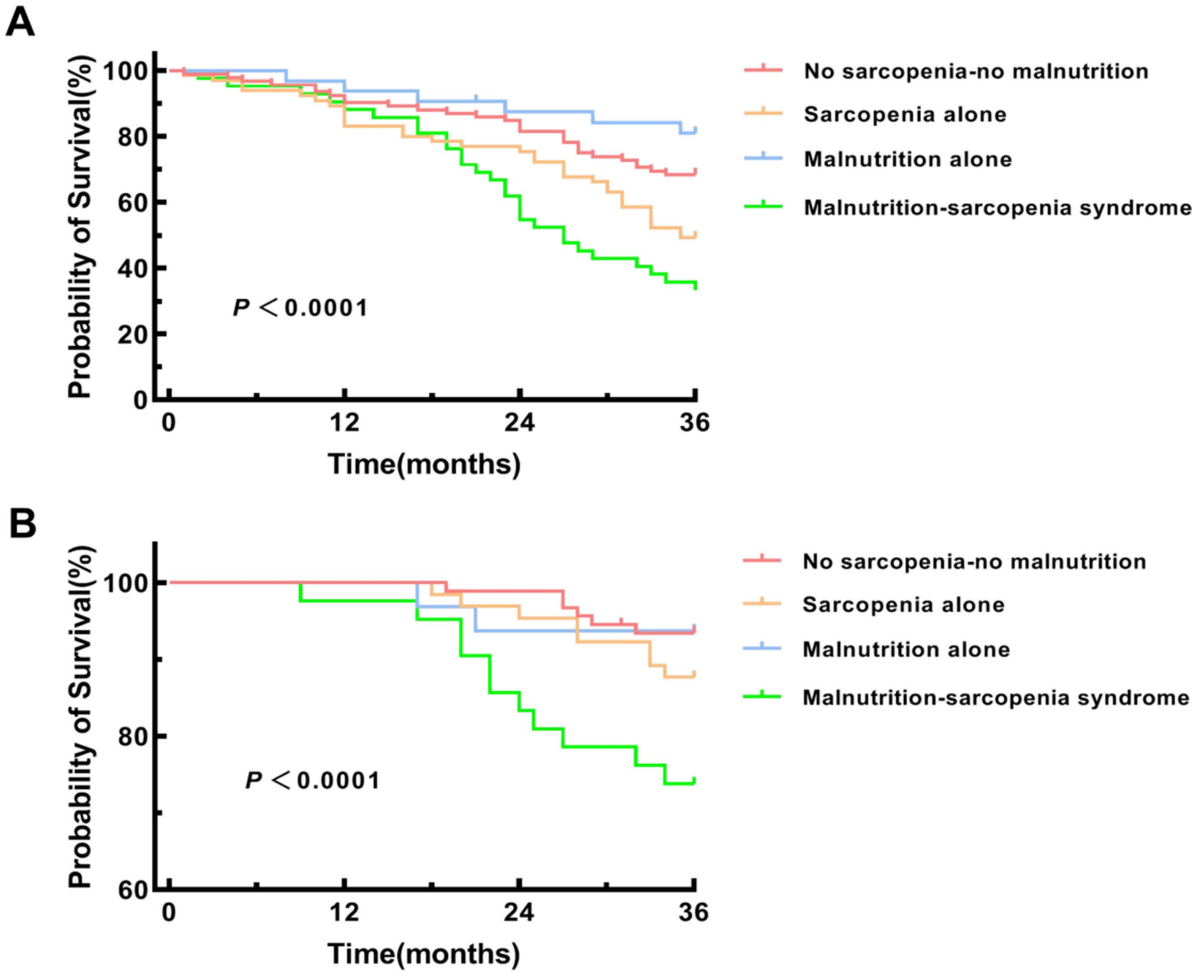
Kaplan–Meier survival curves for MACE and all-cause mortality. **(A)** Kaplan–Meier survival curve for major adverse cardiovascular events (MACE). Pairwise log-rank tests were performed with Bonferroni adjustment (significance level *α* = 0.05/6 = 0.0083) between the MSS group and each of the other three groups. MSS vs. no sarcopenia-no malnutrition group (*p* < 0.001), sarcopenia alone group (*p* = 0.007) and malnutrition alone group (*p* < 0.001). **(B)** Kaplan–Meier survival curve for all-cause mortality. Pairwise log-rank tests were performed with Bonferroni adjustment (significance level *α* = 0.05/6 = 0.0083) between the MSS group and each of the other three groups. MSS vs. no sarcopenia-no malnutrition group (*p* < 0.001), sarcopenia alone group (*p* = 0.006) and malnutrition alone group (*p* = 0.002).

Kaplan–Meier survival analysis was performed to compare MACE incidence and all-cause mortality across four groups: no sarcopenia-no malnutrition, sarcopenia alone, malnutrition alone, and MSS. Pairwise comparisons with Bonferroni adjustment confirmed that the MSS group had a significantly higher risk of both MACE and all-cause mortality compared to each of the other three groups individually (all adjusted *p* < 0.0083) (Figure 6).

### 3.7 Logistic regression analysis of risk factors for AAC and MACE

**Figure 7:**
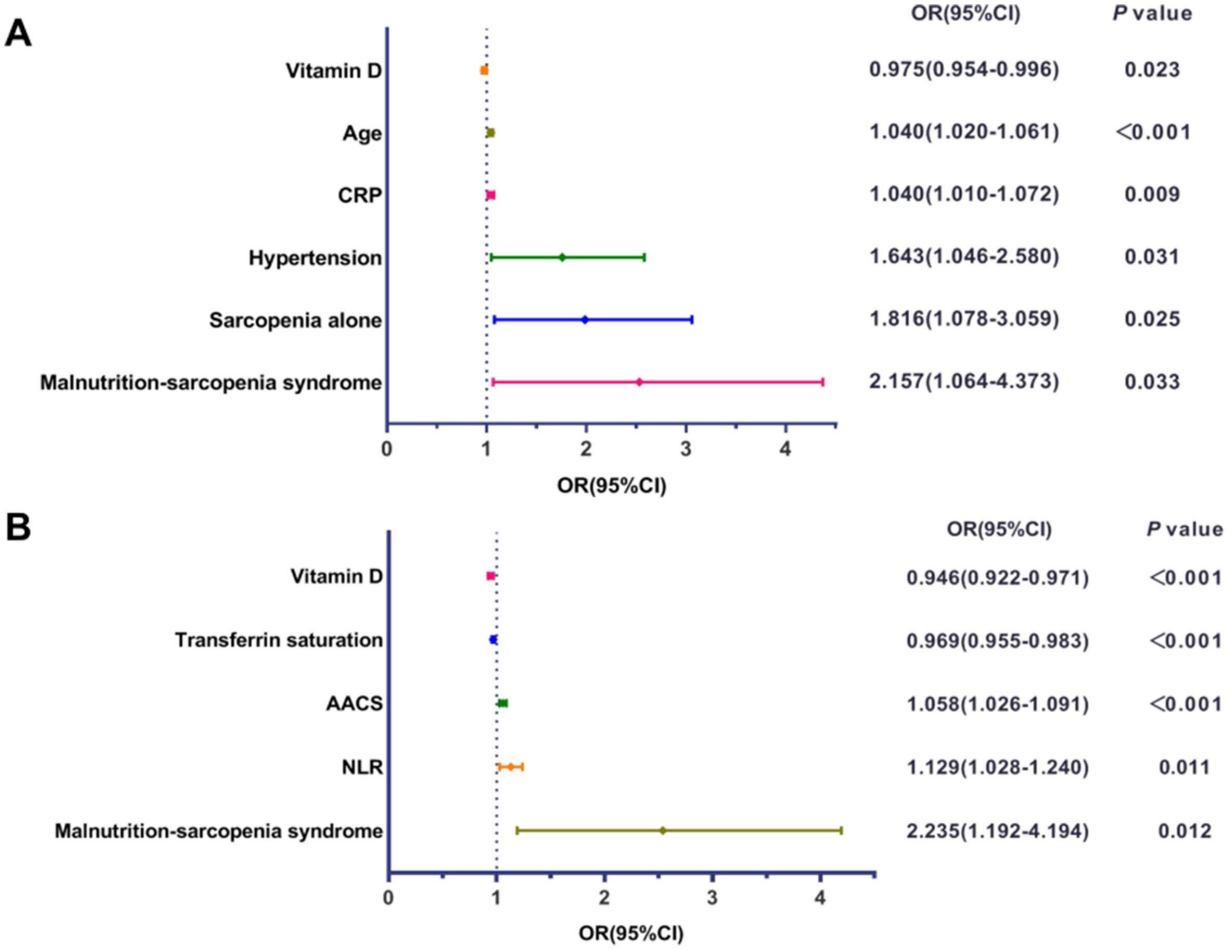
Forest plots of multivariable logistic regression analysis. **(A)** Forest plot displaying the odds ratio (OR) and 95% confidence interval (CI) for independent influencing factors associated with abdominal aortic calcification (AAC). **(B)** Forest plot displaying the OR and 95% CI for independent influencing factors associated with major adverse cardiovascular events (MACE). CRP, C-reactive protein; AACS, abdominal aortic calcification score; NLR, neutrophil-to-lymphocyte ratio.

Multivariable logistic regression analysis was performed to identify independent risk factors for AAC and MACE in HD patients. Covariates included age, gender, BMI, diabetes, hypertension, and CRP, selected based on clinical relevance and previous literature. After adjusting for potential confounders, MSS was found to be an independent risk factor for both AAC and MACE (Figure 7). Patients with MSS had a 2.157-fold higher risk of AAC (OR: 2.157, 95% CI: 1.064–4.373, *p* = 0.033) and a 2.235-fold higher risk of MACE (OR: 2.235, 95% CI: 1.192–4.194, *p* = 0.012) compared to those without MSS. Other risk factors for AAC included age, CRP, hypertension, and sarcopenia alone, while vitamin D was identified as a protective factor. For MACE, transferrin saturation, AAC score, and NLR were identified as risk factors, with vitamin D serving as a protective factor.

### 3.8 Mediation analysis of AAC in the relationship between MSS and MACE

**Figure 8:**
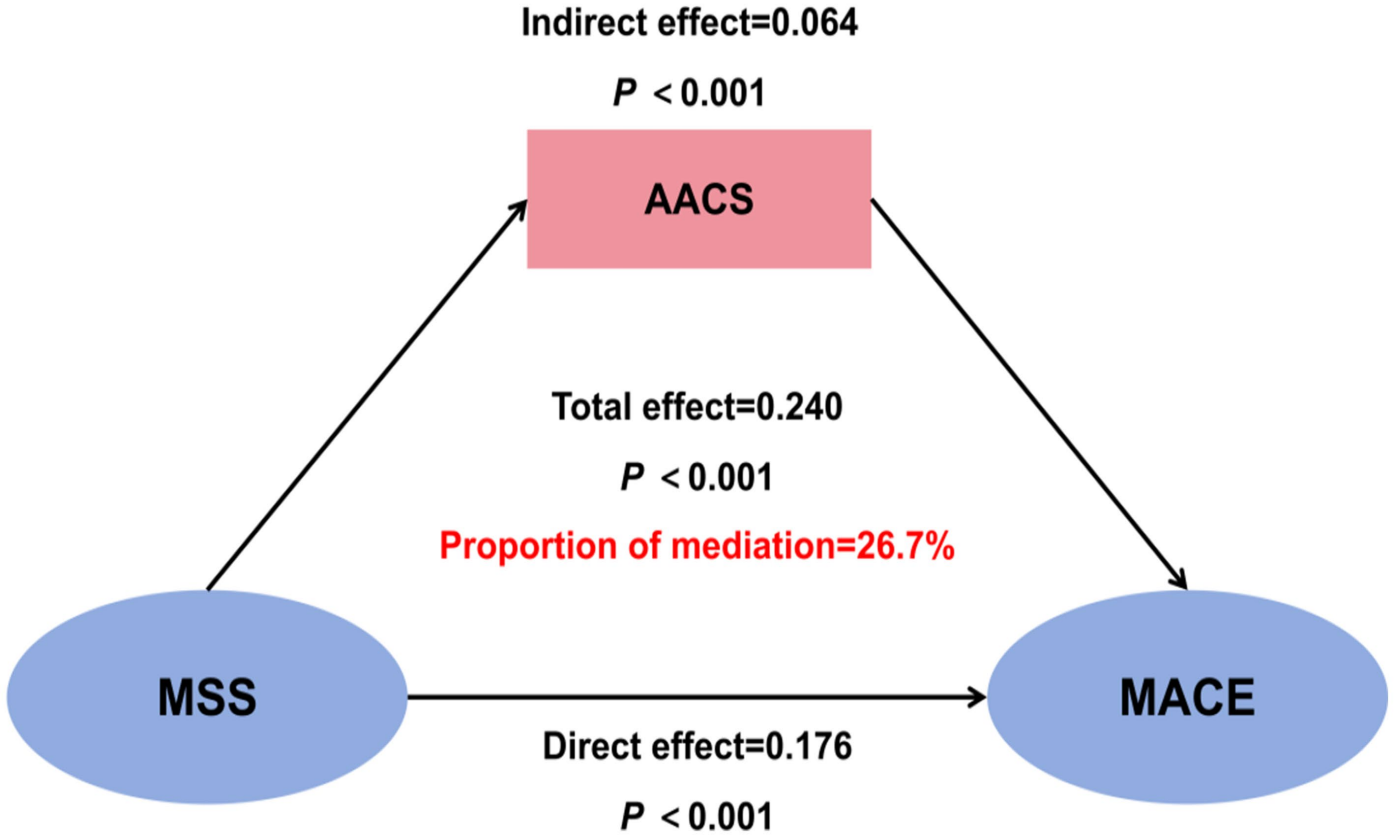
Mediation analysis of abdominal aortic calcification (AAC) in the relationship between malnutrition-sarcopenia syndrome (MSS) and major adverse cardiovascular events (MACE). AACS, abdominal aortic calcification score.

To explore the role of AAC severity in the impact of MSS on MACE, we conducted a mediation analysis. The analysis revealed that MSS had a total effect of 0.240 (95% CI: 0.193–0.421) on MACE, a direct effect of 0.176 (95% CI: 0.110–0.340), and an indirect effect of 0.064 (95% CI: 0.034–0.147) through AAC score, with the proportion of mediation accounting for 26.7% of the total effect (all *p*-values <0.001) (Figure 8).

## 4 Discussion

This study provides the first systematic evaluation of the combined effects of sarcopenia and malnutrition on AAC progression and MACE risk in HD patients. Our prospective cohort analysis yielded three principal findings: (1) the coexistence of sarcopenia and malnutrition, termed MSS, was independently associated with significantly elevated risks of both vascular calcification and cardiovascular events; (2) The AAC burden in patients with MSS was significantly more severe than in those with no sarcopenia or malnutrition and those with malnutrition alone. The comparable severity of calcification between the MSS and sarcopenia-alone groups suggests that sarcopenia might be a predominant risk factor for VC; and (3) the malnutrition-sarcopenia interaction accounted for 26.7% of cardiovascular risk through vascular calcification pathways. Although malnutrition and sarcopenia each predict poor outcomes, their combined impact—and the role of AAC as a mediator—has not been previously quantified in hemodialysis patients. These findings offer novel clinical insights for cardiovascular risk stratification in maintenance dialysis populations.

Sarcopenia has emerged as a critical public health concern, particularly among HD patients. Current diagnostic frameworks incorporate three key elements—muscle strength, mass, and physical performance (21, 25). Although both the European Working Group on Sarcopenia in Older People (EWGSOP) (2019) and AWGS 2019 are widely used diagnostic criteria for sarcopenia, the latter was selected for this study due to the Asian origin of our cohort. 46.3% of participants were diagnosed with sarcopenia, with notable gender disparity (female predominance: 54.6% vs. 40.3%, *p* = 0.002) in our study. Malnutrition prevalence was 32.0% (non-significant gender difference), while sarcopenic patients showed higher malnutrition rates than non-sarcopenic counterparts (39.3% vs. 24.9%, *p <* 0.001), aligning with previous reports (26).

Malnutrition remains prevalent in CKD populations, The European Society for Clinical Nutrition and Metabolism (ESPEN) guidelines (8, 27) also emphasize that due to the lack of a unified and validated assessment method for malnutrition in CKD, its reported prevalence varies considerably across countries and regions—for example, 37% in UK HD patients (28), 58.8% in Brazil (29), and ranging from 11 to 54% in HD patients depending on assessment methodologies, while meta-analyses estimate an approximate prevalence of 42% in HD cohorts (30). The National Kidney Foundation’s Kidney Disease Outcomes Quality Initiative (KDOQI) clinical practice guideline for nutrition in CKD recommend the use of the 7-point Subjective Global Assessment (SGA) as a tool for assessing nutritional status (17). Another validated tool, GNRI, which incorporates serum albumin dynamics and anthropometric parameters, has been validated as a nutritional assessment tool for Japanese CKD patients (24, 31) and demonstrates prognostic utility for mortality prediction in HD populations (14, 32, 33). Although some studies have used lower cutoffs for mortality prediction, the GNRI <92 threshold is widely accepted for identifying malnutrition in HD populations (12, 24, 31). These evidence-based considerations informed our selection of GNRI for malnutrition evaluation.

Both malnutrition (particularly PEW) and sarcopenia are well-established risk factors for multiple complications and mortality in HD patients (34). These conditions frequently coexist and interact through a vicious cycle: malnutrition accelerates muscle loss, while sarcopenia exacerbates nutritional deficits through reduced energy expenditure and decreased dietary intake secondary to physical inactivity (9). This bidirectional relationship creates substantial clinical overlap (13), MSS associated with substantially higher mortality risk compared to either condition alone (35). Our study identified that 18.2% of patients were diagnosed with MSS, a prevalence higher than that in the general population and comparable to the prevalence reported in previous studies of hemodialysis patients (12, 36). We believe that it is necessary to comprehensively evaluate their combined effects rather than consider them as independent entities.

Expanding on the existing body of knowledge, we embarked on the present study to further elucidate the interplay between malnutrition, sarcopenia, and their combined impact on HD patients. Our findings unveiled that individuals with MSS exhibited markedly elevated levels of both CRP and ferritin, which aligns with previous observations (11). These inflammatory biomarkers serve as key indicators of the malnutrition-inflammation-atherosclerosis (MIA) syndrome, a well-documented pathophysiological cascade in HD populations.

Notably, patients who were free from both sarcopenia and malnutrition demonstrated significantly higher serum albumin levels and creatinine levels. These findings reflect a better-preserved nutritional status and muscle mass in this subgroup. Elevated creatinine is a hallmark of ESRD, however, its strong correlation with muscle metabolism (37) and nutritional parameters, independent of dialysis adequacy, highlights the necessity for comprehensive nutritional interventions in HD management protocols.

Additionally, the MSS group had the highest mean age, followed by the sarcopenia alone group, which is consistent with existing studies on HD patients (12, 13). This finding suggests that aging is a significant factor contributing to the development of sarcopenia and the coexistence of sarcopenia and malnutrition. Given the global trend of an aging dialysis population, there is an increasing need to pay attention to this phenomenon.

Survival analyses substantiated these findings, with MSS patients displaying the highest cumulative MACE incidence and poorest overall survival (log-rank *p <* 0.001). While the Kaplan–Meier curves effectively illustrate the unadjusted association between patient groups and outcomes, this analysis does not account for differences in baseline characteristics. The subsequent multivariate regression models provide a more robust, adjusted estimate of the independent risk associated with MSS. Mediation analysis confirmed that AAC severity partially mediated (26.7%) the MSS-MACE association (*β* = 0.064, *p <* 0.001), suggesting malnutrition may potentiate VC in sarcopenic HD patients. The multivariable regression and mediation analyses provide a multi-layered perspective on the pathophysiology linking MSS to adverse outcomes. The differing risk factors for AAC (a structural change) and MACE (a clinical event) are illuminating. AAC was independently associated with traditional factors like age and hypertension, and with factors related to CKD-MBD, such as vitamin D deficiency. Conversely, MACE was more strongly linked to factors indicating functional reserve and acute stress, such as transferrin saturation, and the AAC score itself (underlying vascular burden). The mediation analysis revealed that AAC severity explained only 26.7% of the total effect of MSS on MACE, which suggests that MSS contributes to cardiovascular events through additional pathways beyond vascular calcification. These likely include the profound functional limitation and reduced physiological reserve (making patients more vulnerable to any hemodynamic stress) (12), exacerbated inflammation promoting plaque instability (38, 39), and potential dysregulation of cardiac function directly related to uremic myopathy and metabolic derangements (40).

The combined effects of sarcopenia and malnutrition in HD patients may be explained by several interconnected pathways: (1) Chronic inflammation: Both conditions are associated with a proinflammatory state, creating a vicious cycle that exacerbates the MIA syndrome and accelerates cardiovascular disease progression (5, 40–42). Our findings support this hypothesis, with MSS patients exhibiting significantly elevated CRP and ferritin levels. (2) Vitamin D deficiency: HD patients frequently present with vitamin D deficiency, which contributes to both muscle wasting and vascular calcification. Mechanistically, vitamin D exerts protective effects by (i) upregulating calcification inhibitors (e.g., matrix Gla protein, osteopontin), (ii) suppressing proinflammatory cytokines (43, 44), and (iii) modulating vascular smooth muscle cell calcium sensing via 1,25(OH)₂D₃ (45). (3) Metabolic dysregulation: Additional contributors include insulin resistance, oxidative stress, overactivation of the renin-angiotensin-aldosterone system, and impaired insulin-like growth factor 1 (IGF-1) signaling—all of which may amplify cardiovascular risk in this population (46–48). While these mechanisms require further elucidation, our study underscores the urgent need for clinical interventions targeting MSS in HD patients to mitigate its deleterious cardiovascular consequences.

An intriguing finding worthy of further discussion is the distinct profile of the malnutrition-alone group. These patients, while nutritionally compromised per GNRI, exhibited lower inflammatory markers (CRP, ferritin), less severe vascular calcification, and better survival outcomes compared to the sarcopenia and MSS groups. This suggests that ‘malnutrition’ in isolation may represent a state of primarily caloric and protein deficiency, potentially amenable to nutritional interventions. In contrast, the presence of sarcopenia, either alone or combined with malnutrition (MSS), appears to be a stronger marker of a persistent inflammatory and catabolic state (MIA syndrome) (49), driving more severe vascular pathology and worse clinical outcomes (50). This distinction implies that sarcopenia might be a more critical prognostic indicator than GNRI-defined malnutrition in this population, and that the MSS phenotype identifies those trapped in the most vicious cycle of inflammation, catabolism, and cardiovascular deterioration.

It is important to acknowledge the limitations of our chosen nutritional assessment tool. We used GNRI rather than the Malnutrition-Inflammation Score (MIS) to avoid overlap with sarcopenia diagnostic criteria, which might have omitted some inflammatory aspects. The GNRI incorporates serum albumin, a parameter significantly influenced by systemic inflammation and fluid status, not solely by nutritional intake. This is a deviation from the explicit recommendation by ESPEN against using visceral proteins like albumin for diagnosing malnutrition (8). However, the selection of GNRI was based on its widespread validation and prognostic utility in hemodialysis populations, as evidenced by meta-analyses (22, 32–34). Furthermore, other commonly used tools, such as the MIS and the criteria for PEW, also incorporate albumin, reflecting the challenges in disentangling nutrition from inflammation in this patient group. Consequently, the ‘malnutrition’ identified by GNRI in our cohort, and the associated interventions, likely target a combination of underlying inflammatory drive and true nutritional deficits. However, it is important to note that this study observed standard care rather than a specific nutritional intervention. Future interventional studies are needed to determine whether treating GNRI-defined malnutrition (and by extension, its inflammatory components) can improve cardiovascular outcomes in patients with MSS.

Several limitations should be acknowledged. First, this was a single-center study with a moderate sample size, necessitating external validation. Second, BIA may overestimate lean mass in edema-prone HD patients. Third, malnutrition was not identified according to the KDOQI criteria, but solely through GNRI in this study. GNRI provides a static assessment and may not capture dynamic nutritional changes. Fourth, the 3-year follow-up may be insufficient to fully evaluate long-term VC progression and cardiovascular outcomes. Finally, there is a paucity of RCTs on nutritional or exercise interventions for sarcopenia in dialysis patients, limiting evidence-based recommendations. Furthermore, nutritional support was provided based on clinical need (GNRI <92) rather than random assignment. While our protocol was standardized, the escalation from dietary counseling to ONS and IDPN was contingent upon the severity and persistence of malnutrition, potentially introducing confounding by indication. The observed associations should be interpreted within this context, and future randomized trials are needed to confirm the causal efficacy of targeted nutritional interventions on outcomes in patients with MSS.

## 5 Conclusion

In conclusion, the prevalence of MSS in HD population is 18.2%. Compared with patients who have sarcopenia or malnutrition alone, those with MSS are associated with higher AAC scores and a higher incidence of AAC. Moreover, MSS increases the risk of MACE and mortality in HD patients. These findings highlight the importance of conducting comprehensive nutritional assessments in CKD patients undergoing HD to facilitate early identification of sarcopenia and its overlap with malnutrition. It also underscores the necessity of developing relevant management guidelines to optimize the care of hemodialysis patients and improve their prognosis. Future studies should explore the impact of precision nutritional interventions on adverse outcomes, including CVD.

## Data Availability

The original contributions presented in the study are included in the article/Supplementary material, further inquiries can be directed to the corresponding author.

## Glossary

AAC: Abdominal aortic calcification
ASMI: Appendicular skeletal muscle mass index
BMI: Body mass index
CKD: Chronic kidney disease
CVD: Cardiovascular disease
CRP: C-reactive protein
ESRD: End-stage renal disease
GNRI: Geriatric Nutritional Risk Index
HD: Hemodialysis
IDPN: Intradialytic parenteral nutrition
Kt/V: Urea clearance index
MIA: Malnutrition-inflammation-atherosclerosis
MSS: Malnutrition-sarcopenia syndrome
MACE: Major adverse cardiovascular events
NLR: Neutrophil-to-lymphocyte ratio
ONS: Oral nutritional supplements
PEW: Protein-energy wasting
PTH: Parathyroid hormone
TC: Total cholesterol
TG: Triglycerides
VC: Vascular calcification
