# Supplementary material for: Malnutrition and sarcopenia: a combined risk factor for vascular calcification and cardiovascular events in hemodialysis patients
Source: Front Nutr. 2025 Nov 14;12:1625935. doi: 10.3389/fnut.2025.1625935 (PMC12661162; doi:10.3389/fnut.2025.1625935)
Supplement: Supplementary file 1 [file Table_1.docx]

Supplementary Table 1: Standardized Protocol for Nutritional Support in Malnourished Hemodialysis Patients

| Component | Intervention Details | Initiation & Dosing Criteria | Administration & Monitoring |
| --- | --- | --- | --- |
| Dietary Counseling | Individualized counseling by renal dietitian to achieve energy intake of 30-35 kcal/kg/day and protein intake of 1.2-1.4 g/kg/day (≥50% high biological value). | Initiated for all patients with GNRI <92. Dosing personalized based on body weight, comorbidities (e.g., diabetes), and dietary intake assessment. | Monthly 30-min sessions. Adherence and intake monitored using 3-day dietary diaries every 3 months. |
| Oral Nutritional Supplements (ONS) | Renal-specific ONS (e.g., Nepro®, Suplena®). ~475 mL per unit provides ~475 kcal, ~20 g protein, low electrolyte content. | Added if dietary intake remains inadequate (<75% of targets) after 1 month of counseling. Dose: 1 unit/day, increased to 2 units/day if tolerated and targets still not met. | Administered orally on non-dialysis days or post-dialysis. Adherence monitored by counting returned empty bottles weekly. Compliance rate calculated as (consumed units / prescribed units) * 100%. |
| Intradialytic Parenteral Nutrition (IDPN) | 500 mL solution containing: 250 mL of 20% lipid emulsion, 250 mL of 10% amino acids, and 100 g of glucose. Provides ~750 kcal and 25 g protein per session. | Reserved for patients with severe malnutrition (GNRI <82) or those unable to tolerate adequate oral/enteral intake. Dose: Administered during each hemodialysis session. | Administered IV via the dialysis blood line over the entire 4-hour session. Adherence was 100% as it was directly administered by clinical staff during HD. |
| Overall Compliance |  |  | The overall compliance rate for the ONS component was 78.4%. The IDPN component had 100% compliance. |
